# The Role of B Cells and Antibodies in Multiple Sclerosis, Neuromyelitis Optica, and Related Disorders

**DOI:** 10.3389/fimmu.2019.00201

**Published:** 2019-02-08

**Authors:** Silke Häusser-Kinzel, Martin S. Weber

**Affiliations:** ^1^Institute of Neuropathology, University Medical Center, Göttingen, Germany; ^2^Department of Neurology, University Medical Center, Göttingen, Germany

**Keywords:** B cells, multiple sclerosis, central nervous system, antigen-presenting cell, cytokine secretion, regulatory B cells, anti-CD20 therapy, neuromyelitis optica-spectrum disorders

## Abstract

Our pathophysiological concept of the most common central nervous system demyelinating disease, multiple sclerosis, strikingly evolved by recent discoveries suggesting that B lymphocytes substantially contribute in its initiation and chronic propagation. In this regard, activated B cells are nowadays considered to act as important antigen-presenting cells for the activation of T cells and as essential source of pro-inflammatory cytokines. Hereby, they create a milieu in which other immune cells differentiate and join an orchestrated inflammatory infiltration of the CNS. Without a doubt, this scientific leap was critically pioneered by the empirical use of anti-CD20 antibodies in recent clinical MS trials, which revealed that the therapeutic removal of immature and mature B cells basically halted development of new inflammatory flares in otherwise relapsing MS patients. This stabilization occurred largely independent of any indirect effect on plasma cell-produced antibody levels. On the contrary, peripherally produced autoantibodies are probably the most important B cell component in two other CNS demyelinating diseases which are currently in the process of being delineated as separate disease entities. The first one is neuromyelitis optica in which an antibody response against aquaporin-4 targets and destroys astrocytes, the second, likely distinct entity embraces a group of patients containing antibodies against myelin oligodendrocyte glycoprotein. In this review, we will describe and summarize pro-inflammatory B cell properties in these three CNS demyelinating disorders; we will however also provide an overview on the emerging concept that B cells or B cell subsets may exert immunologically counterbalancing properties, which may be therapeutically desirable to maintain and foster in inflammatory CNS demyelination. In an outlook, we will discuss accordingly, how this potentially important aspect can be harnessed to advance future B cell-directed therapeutic approaches in multiple sclerosis and related diseases.

## Introduction

The fulminant clinical success of anti-CD20 antibodies in the treatment of multiple sclerosis (MS) and neuromyelitis optica-spectrum disorders (NMO-SD) raised awareness that beside T cells, B cells play a decisive role in their initiation, and propagation. Here, the rather immediate benefit of anti-CD20 therapy has been mainly attributed to the extinction of B cells from the blood, but even more so from immunological relevant organs, such as lymph nodes and spleen (1). In these peripheral compartments, B cells interact with other immune cells after encountering antigen, promote their differentiation and in turn undergo expansion and maturation themselves (2). In NMO-SD, this peripheral B cell activation results in a highly relevant antibody response against CNS antigen. Consequently, most investigations focused on elucidating mechanisms by which B cells contribute to the pathogenesis of MS and NMO-SD in the periphery. These studies revealed that beyond antibody production, cellular properties of B cells such as antigen presentation and cytokine production shape the response of other immune cells such as T cells and myeloid cells both in a pro-inflammatory, but also in a regulatory manner. Besides these properties in the periphery, B cells and their antibodies probably play an important role within the CNS, which may however substantially differ between MS and NMO-SD.

## B Cells Contribute as Antigen-Presenting Cells to the Activation of T Cells

**Figure 1:**
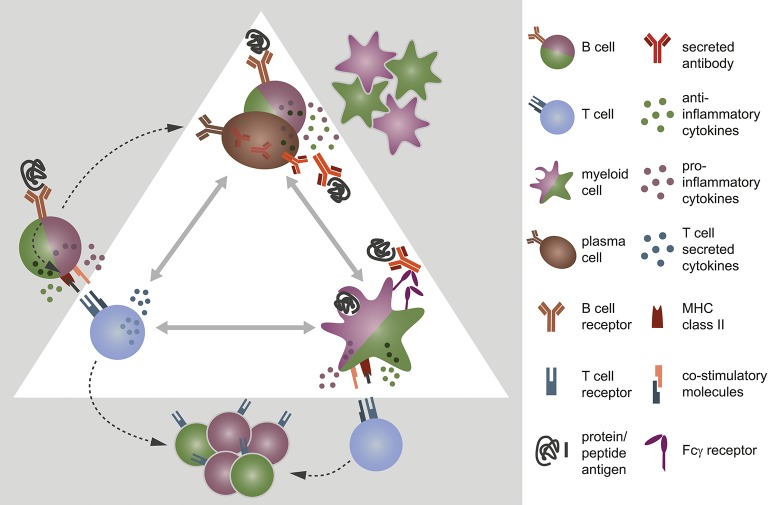
B cells, T cells, and myeloid cells shape each other's immune response via direct interaction and/or secretion of cytokines. **(A)** B cells encounter protein antigens specifically via their B cell receptor and present linearized peptides bound to the major histocompatibility complex (MHC) class II to T cells. Thereby, they act as efficient antigen-presenting cells and control the differentiation of T cells by the density of co-stimulatory molecules on their cell surface and the cytokine milieu they provide. In turn, this interaction fosters **(B)** the differentiation of B cells into antibody-producing plasma cells and memory B cells. B and plasma cells secrete pro- and anti-inflammatory cytokines, which affect the expression of co-stimulatory molecules and the production of chemokines/cytokines by myeloid antigen-presenting cells. Vice versa, myeloid cells have an impact on B cell activity through the secretion of distinct cytokines and chemokines. **(C)** Myeloid antigen-presenting cells, such as monocytes, macrophages, and dendritic cells internalize antigen randomly or opsonized antigen specifically via Fcγ receptors, process them, and present the linearized peptides via MHC class II to T cells. They are able to induce both pro- and anti-inflammatory T cells, controlled by the expression density of co-stimulatory molecules on myeloid APC and their distinct secretion of cytokines.

B cells are professional antigen-presenting cells (APC): they recognize even low concentrations of antigens specifically and constitutively express major histocompatibility complex (MHC) class II and co-stimulatory molecules. This enables B cells to prime T cells and in turn induces their own differentiation into memory cells and antibody-producing plasma cells (Figure 1A). In contrast to myeloid APC, which randomly ingest peptides, B cells are capable of specifically recognizing, and internalizing natively folded “conformational” protein antigens via their B cell receptor. Subsequently, B cells process these structures to short linearized peptides and present it to antigen-specific T cells via MHC class II molecules. Thus, B cells are most efficient APC when they share antigen recognition with responding T cells (3). In genetically-altered mice containing myelin specific B and T cells, the mere coexistence of these cells induces a spontaneous form of experimental autoimmune encephalomyelitis (EAE) (4, 5), a commonly used murine model for human inflammatory CNS demyelinating disorders. In the very same model, the selective ablation of MHC class II on B cells renders mice resistant to disease induction (6), showing their substantial contribution as APC to this model. However, efficient priming of naïve T cells does not only rely on peptide presentation via MHC class II, but furthermore requires the ligation of co-stimulatory molecules, such as CD40, CD80, and CD86. The quality of these signals in conjunction with soluble factors shapes the emerging effector T cell type. While for instance a strong cell-cell contact via CD40 on B cells and CD40 ligand (CD40L) on T cells is necessary for the generation of pro-inflammatory T cells, a weaker molecular contact induces rather regulatory T cell functions and a complete block of CD40-CD40L interaction even prevents EAE (7, 8). In line with these findings, B cells of active MS patients compared to controls express increased amounts of CD40 together with higher level of MHC class II and CD80 (9, 10) suggesting that they harbor an enhanced APC capacity. Furthermore, peripheral as well as CNS B cells exhibit signs of chronic activation with a shift toward antigen-experienced memory B cells (11, 12) pointing toward an active involvement of B cells in MS pathogenesis. This assumption is further corroborated by functional studies which revealed that in a subgroup of relapsing-remitting MS patients, B cells were capable of initiating proliferation, and interferon-gamma (IFN-γ) secretion of potentially pathogenic CD4^+^ T helper (Th)1 cells *ex vivo* (13). In summary, these findings point toward an active involvement of B cells in the pathogenesis of MS, potentially by activating CNS-infiltrating T cells that in turn drive inflammation in brain and spinal cord.

## B Cells Secrete Pathogenic, But Also Regulatory Cytokines, Which Control Other Immune Cells

Besides being equipped with molecules required for direct cell-cell contact, B cells provide a variety of cytokines for inter-cell signaling. This is important as T cell activation does not only rely on the strength of co-stimulatory signals, but furthermore the cytokine milieu provided by the presenting cell (Figure 1B). For instance, interleukin (IL)-6 secreted by B cells fosters the differentiation of Th17 cells, while it prevents the generation of regulatory T cells (14, 15). Thus, in a B cell dependent EAE setting, B cell-restricted IL-6 deficiency diminished the Th17 response and ameliorated the disease severity (6, 16). B cells isolated from the blood of MS patients though exhibit an abnormal pro-inflammatory cytokine profile when compared to healthy controls. They secrete elevated amounts of IL-6, lymphotoxin alpha and tumor necrosis factor alpha (TNF-α), and produce less anti-inflammatory IL-10 (11, 16). The observation that these abnormalities were apparent upon polyclonal stimulation suggests that not only autoreactive B cells but rather the B cell pool at large is deregulated in individuals with MS (11, 17). Moreover, MS patients showed an increased frequency of memory B cells that co-express the pro-inflammatory cytokines granulocyte-macrophage colony-stimulating factor (GM-CSF), IL-6, and TNF-α. In the small MS cohort investigated, therapeutic removal of B cells including the latter memory B cell subpopulation resulted in a diminished pro-inflammatory IL-6 response by macrophages in a GM-CSF-dependent manner (18). An observation that points toward an inflammation-promoting potential of B cells in MS. However, a similar investigation aiming to assess the activation of myeloid APC in blood before and after therapeutic B cell removal in MS and NMO patients did not reveal such uniform results. Here, the macrophages of the study participant showed similar TNF-α secretion before treatment initiation, but varied widely after anti-CD20 therapy (19). This suggests that B cell depletion had a differential effect on the activation of myeloid cells in individual patients, with either pro-inflammatory, or anti-inflammatory outcomes (Figure 1C). Moreover, it indicates that in a subgroup of MS patients, B cells may exert immune regulatory functions prior to their therapeutic removal. Indeed, B cells are not only a relevant source of pro-inflammatory, but moreover of anti-inflammatory cytokines: while antigen-activated B cells mostly secrete pro-inflammatory ones, antigen-naïve B cells, plasmablasts, and plasma cells produce relevant amounts of anti-inflammatory IL-10, IL-35, and transforming growth factor beta (TGF-β). In the context of EAE, adoptive transfer of IL-10-secreting B cells for instance suppressed disease (20), while B cell-restricted abrogation of IL-10 or IL-35 augmented its severity. Moreover, both B cell-derived IL-10 and IL-35 were required for physiological recovery from an acute disease flare (21, 22), and the presence of B cell-secreted TGF-β limited the induction phase of EAE (23). In all of these studies, augmented EAE severity went along with an increased number of differentiated, pro-inflammatory Th1, and Th17 cells, suggesting that anti-inflammatory cytokines secreted by B cells were required to limit the pathogenic T cell response during EAE. In humans, similar regulatory B cell properties have been described (24) and are assumed to be impaired in MS patients (11). However, further research is required to validate this assumption and to ascertain whether regulatory B cells are equally relevant in MS as they are in EAE. If this proves true however, future therapies should aim to maintain or restore regulatory B cell functions, while targeting pro-inflammatory properties selectively; an issue that currently available therapies cannot address (25, 26). In this context, a promising approach may be the inhibition of Bruton's tyrosine kinase (Btk), an enzyme that is present in B cells, and innate immune cells, such as myeloid APC, but not in T cells. B cells require Btk for proper B cell receptor signaling, where it rather modulates the signal responsiveness, than turning it on or off (27). Thus, its inhibition does not deplete B cells, but presumably lowers their response to B cell receptor stimuli (28). In this way, Btk inhibition is assumed to foster the induction and maintenance of tolerogenic B cells, while it counteracts their antigen-mediated pro-inflammatory activation (29–31). In mice with collagen-induced arthritis and in a murine lupus model, both autoimmune disorders with pathogenic B cells involvement, an orally applied Btk inhibitor reduced the amount of circulating autoantibodies and inhibited the development of disease (32), showing its ability to limit a pathogenic B cell response. In MS, first phase II clinical trials testing evobrutinib (ClinicalTrials.gov Identifier: NCT02975349), an orally applied, highly selective Btk inhibitor, significantly reduced the number of new enhancing T1, and new or enlarging T2 lesions when compared to placebo (ECTRIMS Online Library. Montalban X. Oct 12, 2018; 232075). These preliminary results suggest that a monotherapy aiming to inhibit Btk can be promising in MS. Moreover, Btk inhibition may be suitable as maintenance therapy after initial anti-CD20-mediated B cell depletion to avoid recurrence of pathogenic B cells.

## B Cells Differentiate Into Antibody-Producing Plasma Cells

As mentioned before, the process of antigen presentation does not only activate the responding T cell but in turn induces the proliferation of the presenting B cell and its subsequent differentiation into memory cells and antibody-producing plasma cells. Hence, the presence of persisting oligoclonal immunoglobulins (Ig) termed oligoclonal bands (OCB) in the cerebrospinal fluid (CSF) of most MS patients (33–35) can be construed as a first evidence of the pathogenic activation of B cells in MS. More detailed investigation revealed that these intrathecal Ig were most likely produced by plasma cells within the CSF as the CSF Ig proteome and the Ig transcriptome of CSF-located B cells matched with each other (36). In addition, intrathecal B cells show signs of somatic hypermutation and clonal expansion (37, 38) pointing toward a germinal center-like reaction with antigen-driven affinity maturation within the CNS. However, there is new evidence that these terminally differentiated B cells in the CSF were not solely a product of intrathecal maturation, but can cross the blood-brain barrier and interact with the peripheral immune system (39–42). How this migration though influences the maturation of intrathecal B cells in detail and whether it affects the peripheral B cell response is not yet fully understood. Up to now, the expression pattern of OCB in the CSF do not have an apparent correlate in the blood, indicating that despite the ability of B cells to exchange, antibody-secreting plasma cells mainly accumulate within the CNS of MS patients. However, the pathogenic relevance of these CNS-located B cells and their products for the pathogenesis of MS is still controversially discussed. The presence of co-localizing Ig and complement depositions in ongoing MS lesions (43) suggests that autoantibodies are involved in CNS injury. A assumption that has been further fueled by studies demonstrating that antibodies isolated from the CSF of MS patients induce axonal damage and complement-mediated demyelination when applied to human CNS tissue *ex vivo* or *in vitro* (44, 45). Nevertheless, the particular antigen(s) recognized by these antibodies are still unclear (46). Reiber et al. (47) for instance claimed that OCB of MS patients were mostly directed against CNS-unrelated antigens, such as rubella, measles, and varicella zoster virus indicating an unspecific “bystander” activation of B cells. Others however proposed autoantibodies against CNS structures, such as myelin, astrocytes, and neuroglial cells to be part of this intrathecal humoral immune response. They report that OCB of MS patients contain autoantibodies against myelin basic protein (48), myelin-associated lipids (49), contactin-2 (50), and KIR4.1 (51). However, the variety of proposed antibody specificities and the fact that some of the aforementioned findings were not easily reproducible by other laboratories (52–54) possibly reflect the complexity of MS pathogenesis. Alternatively, it suggests that MS may consist of multiple disease entities with distinct disease driving mechanisms. In fact, the first clinical variant of MS, which has been separated from the “core disorder” was NMO based on the discovery of anti-aquaporin (AQP)4 autoantibodies in the patients' blood (55, 56). AQP4 is a water channel found both in peripheral organs such as the kidney (57) as well as in the CNS (58). There it is mainly expressed on the end feet of astrocytes (59, 60), most densely in the optic nerve and spinal cord where astrocytes and oligodendrocytes are in close proximity (61). Hence, these are the regions where NMO lesions predominantly occur. Since AQP4 is not expressed on oligodendrocytes themselves (58), astrocytes are suggested to be the main target in NMO (62, 63). Corroborating this notion, active NMO lesions contain areas of co-localizing Ig and complement depositions with a vast loss of AQP4 and glial fibrillary acid protein immunoreactivity that points toward an antibody-mediated destruction of astrocytes. Older lesions however show in addition a reduced number of oligodendrocytes and extensive demyelination of gray and white matter (56, 64, 65) indicating that demyelination occurs secondarily in later stages of the disease as a result of the preceding astrocyte loss. Hence, NMO is nowadays recognized as an autoimmune astrocytopathy (66) which is, at least in part, mediated by autoantibodies against AQP4. Interestingly, anti-AQP4 antibody titer are relatively low or even absent in the CSF of NMO patients even when the corresponding antibody titer in the blood are high (67). Furthermore, only 15–30% of NMO patients have OCB in the CSF, which in addition mostly disappear with disease progression (68). These findings together suggest that in NMO, B cells are in the first place activated outside the CNS resulting in a pronounced humoral immune response against AQP4 in the periphery. However, new data indicate that also in NMO patients, similar to MS, B cells exchange across the blood-brain barrier resulting in the presence of AQP4-specific B and plasma cells both in the blood and the CSF (69). Nevertheless, the particular trigger(s) of these astrocyte-directed attacks and the exact sequence of B cell activation including the circumstances under which AQP4-directed B cells and/or antibodies gain access to the CNS to induce lesion formation are not fully understood. Despite these pending mechanistic issues, the diagnosis of NMO is nowadays closely tied to the presence of antibodies against AQP4. However, some patients with clinical features suggestive for NMO do not have detectable anti-AQP4 antibody titers. Instead, about a third of them produce antibodies against myelin oligodendrocyte glycoprotein (MOG) in the blood (70–72). MOG is a transmembrane protein expressed on the outermost lamella of the myelin sheath and the surface of oligodendrocytes (73). Its extracellular localization and its lack of expression in the thymus renders MOG a plausible target for autoimmune responses (74, 75). Patients with autoantibodies against MOG have a severe disease course with high relapse rates, strong brainstem, and spinal cord involvement and do hardly respond to several disease-modifying treatments (54). Evaluation of their CSF and histological analysis of biopsy/autopsy tissue revealed no astrocytopathy, but myelin damage as primary injury in the CNS (1, 54, 76–78). Similar to classical NMO, OCB occur only occasionally (79), and anti-MOG antibodies can be found in the serum, but not in the CSF (80, 81).

## Pathogenic Involvement of B Cells and Their Products in the Periphery and Within the CNS

The occurrence of a peripheral humoral immune response against CNS antigen is the most striking similarity between patients with anti-AQP4 and anti-MOG antibodies. It delineates them distinctly from MS patients, which show an accumulation of Ig in the CSF, but have no apparent reflection of these antibody patterns in the blood. However, the pathogenic role of these autoantibodies outside the CNS is still elusive. In mice, it has been demonstrated that peripheral anti-MOG antibodies foster the activation of encephalitogenic T cells in the periphery by opsonization of otherwise unrecognized traces of CNS antigen, which results in the induction of EAE (82, 83). How these endogenous CNS antigens though reach the periphery is uncertain, but presumably by being drained from the CNS to peripheral lymph nodes along lymphatic vessels (84). Even though it is not yet proven that this mechanism is of relevance for the human condition, it is conceivable as antibodies isolated from anti-MOG antibody positive patients were capable of opsonizing human MOG (83). Furthermore, traces of myelin have been found in cervical lymph nodes of MS patients as well as healthy controls (85, 86) indicating that also in humans, CNS structures can be made accessible to the peripheral immune system by this route. Consequently, it includes the possibility that CNS antigens are recognized and opsonized by CNS-directed autoantibodies in the periphery. Overall, these findings suggest that anti-AQP4 antibody positive NMO as well as MOG antibody-associated disease is primarily driven by a pathogenic B cell activation in the periphery resulting in the generation of antibody-producing plasma cells, again in the first place in the periphery. In contrast, in MS, B cells probably exert their pathogenic properties both in the periphery as well as within the chronically inflamed CNS itself, but most probably independent of CNS-specific peripheral antibodies. After activation, B cells migrate through blood or lymph vessels into peripheral lymphoid organs, where they undergo full activation and maturation. Currently available immune-modulating MS therapies are very efficient in targeting these peripheral immune cells, but have only little or no access to the CNS-compartmentalized cells (87, 88). New concepts though suggest that two, probably independent, inflammatory processes drive CNS injury in MS, and potentially involve B cells: on the one hand, *de novo* infiltration of immune cells from the periphery into the CNS that correspond with focal inflammation, MRI-detectable lesions, and relapses. On the other hand, chronic progression supposedly driven by CNS-intrinsic inflammation that is promoted by CNS-resident immune cells in conjunction with CNS-trapped leukocytes (89). The first mechanism is premised on abnormally activated immune cells that migrate from lymphatic tissue, the location of their priming, across the blood-brain barrier into the CNS. There, these leukocytes are assumed to reactivate and contribute to the injury of axons and glial cells (90–92) forming focal lesions. These lesions are typically located perivascular and contain T cells, monocytes, B, and plasma cells (93). Since anti-CD20-mediated B cell depletion is highly efficient in preventing the formation of such focal CNS lesions, its assumed therapeutic efficiency is mainly based on the abrogation of the aforementioned cellular B cell properties in the periphery, and within the perivascular space (94). Chronic progression in contrast is characterized by gradual expansion of consisting lesions with myelin-containing macrophages at the lesion border, gray, and white matter atrophy as well as diffuse aberrant inflammation of the normal-appearing white matter (95, 96). In progressive MS, this cortical demyelination has been further associated with B cell-rich structures in the meninges (97, 98) as well as with plasma cell accumulation in experimental CNS inflammation (99). These findings point toward a gradual shift of disease-driving B cell functions from the periphery to the CNS with disease progression. Furthermore, they indicate that B cells may be involved—directly or indirectly—in cortical injury. An observation that is further corroborated by the findings of Lisak et al. (100) demonstrating that secretory products independent of antibodies and multiple cytokines produced by B cells of progressive MS patients are cytotoxic to oligodendrocytes and neurons (101). In line with these results, it is not surprising that even though anti-CD20 is highly efficient in limiting the formation of new CNS lesions, it does not entirely stop chronic progression. This further strengthens the assumption that chronic CNS injury in MS is not primarily caused by *de novo* infiltrating immune cells, but by an established CNS-compartmentalized inflammation, which results in a CNS-autonomous immune response over time.

## Conclusion

Current research indicates that in MS, B cells contribute to the formation of relapses as well as to the progression of the disease independent of *de novo* CNS infiltration. In contrast, in NMO and anti-MOG antibody-associated demyelination, a peripherally generated CNS-targeting antibody response is suggested to be the main disease driver. Accordingly, these delineating disease entities may require MS-independent therapeutic strategies, a concept that is currently evolving. Thus, therapies targeting distinct aspects of NMO-relevant B cell functions such as plasma cell differentiation and complement fixation are currently under evaluation. First trials showed promising results for the treatment with tocilizumab, an therapeutic antibodies against IL-6 receptor (102, 103), and eculizumab, an complement component 5-specific antibody (104). Besides these pathogenic B cell properties, B cells, or B cells subsets likely exert a therapeutically desirable regulatory function in either disease, limiting tissue inflammation as well as pro-inflammatory activation of other immune cells. Accordingly, one of the prime challenges for the long-term targeting of B cells in MS and related demyelinating diseases will be to delineate and specifically target pathogenic B cell properties by novel strategic concepts, such as the selective depletion of differentiated B cells, interference with their activation or ablation of a disease-driving antibody response.

## Author Contributions

SH-K drafted the manuscript and prepared the figure. MW drafted, wrote, and finalized the manuscript.

### Conflict of Interest Statement

The authors declare that the research was conducted in the absence of any commercial or financial relationships that could be construed as a potential conflict of interest.
